# Synthesis of Double-Shell Hollow TiO_2_@ZIF-8 Nanoparticles With Enhanced Photocatalytic Activities

**DOI:** 10.3389/fchem.2020.578847

**Published:** 2020-10-23

**Authors:** Ning Fu, Xue-chang Ren

**Affiliations:** ^1^School of Environmental & Municipal Engineering, Lanzhou Jiaotong University, Lanzhou, China; ^2^Gansu Environmental Monitoring Center, Lanzhou, China

**Keywords:** hollow nanoparticles, metal-organic frameworks, TiO_2_, hollow, photocatalysis

## Abstract

Synthesis of semiconductor-MOF heterostructure photocatalysts has attracted considerable attention for their thermal stability, controllable crystallinity, and enhanced photocatalytic activity. In this work, the hollow nanostructure of anatase TiO_2_ was prepared by etching SiO_2_ from core–shell SiO_2_@TiO_2_ nanoparticles. ZIF-8, one of the metal-organic frameworks (MOFs), was hybrid synthesized on the surface of hollow TiO_2_ and formed double-shell hollow nanoparticles. The photocatalytic activity of the double-shell hollow TiO_2_@ZIF-8 nanoparticles toward methylene blue (MB) under UV light irradiation was processed, and the highest photocatalytic efficiency of 99.1% was shown compared with TiO_2_ and SiO_2_@TiO_2_ nanoparticles. This study suggests a promising approach to achieve an advanced photocatalytic performance toward dye degradation using MOFs for the surface engineering of semiconductors.

## Introduction

TiO_2_, one of the most important semiconductor photocatalysts, has been widely used for removing organic pollutants due to its high photocatalytic activity, low cost, and chemical stability (Waldmann and Paz, 2016; Sun et al., 2019). However, the large band gap, high recombination rate of electron–hole pairs, and only utilization of UV light limit the practical photocatalytic applications of TiO_2_ (Nakata and Fujishima, 2012; Schneider et al., 2014; Shen et al., 2018). It has been reported that the photocatalytic performance of TiO_2_ can be significantly influenced by different morphologies such as nanotubes (Nakata et al., 2011), nanorods (Wang et al., 2007), nanosheets (Aoyama et al., 2012), and hollow spheres (Liu et al., 2009). Among these structures, TiO_2_ hollow spheres show enhanced photocatalytic performance because of their low density, high specific surface area, good surface permeability, and high light-trapping efficiency (Wu et al., 2012; Pan et al., 2014). In addition, the hollow TiO_2_ structure can reflect UV light within the hollow sphere interior and enhance light harvest to improve the photocatalytic property (Shen et al., 2012; She et al., 2013).

Recently, metal-organic frameworks (MOFs) have received intense interests for applications in gas separation, catalysis, and drug delivery due to its potential advantages such as high surface area, large and well-ordered porous structures, and structure designability (Wang and Wang, 2015; Wang et al., 2019). In addition, some MOFs show the potential properties of photocatalysts for catalytic degradation of organic pollutants under UV-visible irradiation (Jiang et al., 2013). However, the catalytic efficiency of MOFs is lower than that of the traditional semiconductors (TiO_2_, CdS, and ZnO) because of low charge separation efficiency (Alvaro et al., 2007; Lee et al., 2009; Wang et al., 2015). In this regard, MOFs are designed by combining with other traditional semiconductors to form heterostructure of photocatalysts for enhancing the photocatalytic efficiency.

Zeolitic imidazolate framework-8 (ZIF-8), as one kind of MOFs, is constructed by 2-methylimidazole ligands and Zn(II) ions, which exhibits higher thermal and chemical stability than other MOFs, and is selected to degrade organic pollutants under UV light irradiation (Huang et al., 2014; Jing et al., 2014; Yang et al., 2015). Studies have shown that the heterojunction of ZIF-8 and TiO_2_ show better photocatalytic efficiency than that of TiO_2_ and ZIF-8 due to the higher surface area and suppression of electron–hole recombination. Liu et al. integrated nanosized ZIF-8 particles on mesoporous TiO_2_ to reduce Cr(VI) by photocatalysis and exhibited remarkable photocatalytic activity than TiO_2_ and ZIF-8 (Liu et al., 2017). Li et al. synthesized TiO_2_ nanospheres growing uniformly on the surface of ZIF-8 and used them for degrading tetracycline (Li et al., 2020). Jia et al. integrated N and F co-doped TiO_2_ nanotubes with ZIF-8 for enhanced photo-electrocatalytic degradation of sulfamethazine. Zeng et al. studied the TiO_2_/ZIF-8 hybrid photocatalysts by coating ZIF-8 on TiO_2_ nanofibers for photodegradation of rhodamine B (Zeng et al., 2016).

However, few works focus on hollow TiO_2_ spheres decorated with ZIF-8 for photocatalytic performance. Zhang et al. synthesized hollow TiO_2_ nanoparticles with ZIF-8 decorated for photocatalytic hydrogen generation under UV light irradiation, and the double-shell hollow TiO_2_@ZIF-8 nanospheres showed better photocatalytic activities in hydrogen generation than TiO_2_ and ZIF-8 (Zhang et al., 2018). At present, there were few works about double-shell hollow TiO_2_@ZIF-8 nanoparticles for photodegradation dyes. In this work, the hollow TiO_2_ nanoparticles were prepared by etching SiO_2_ from core–shell SiO_2_@TiO_2_ nanoparticles, and then ZIF-8 was synthesized on the surface of hollow TiO_2_ nanoparticles and formed the double-shell hollow nanoparticles. The photocatalytic activities of the synthesized nanoparticles for photodegradation of MB under UV irradiation were processed. This work illustrated the strategy of enhancing photocatalytic activity by modifying hollow TiO_2_ nanoparticles with a high surface area of ZIF-8 to improve adsorption and charge transfer efficiency.

## Materials and Methods

### Synthesis of SiO_2_ Core Spheres

The SiO_2_ core spheres were prepared using the Stöber method according to the reported paper (Lin et al., 2015). Firstly, 10 mL of tetraethyl orthosilicate (TEOS) was added to 90 mL of ethanol under continuous stirring to form solution A; meanwhile, 20 mL of water and 10 mL of aqueous ammonia were added to 70 mL of ethanol under vigorous stirring to form solution B. Then, solutions A and B were mixed under continuous stirring for 3 h at 40°C. Finally, the products were centrifuged at 4,000 rpm and washed twice with methanol and once with water. The final SiO_2_ core spheres were obtained by drying at 70°C for at least 20 h.

### Synthesis of Core–Shell SiO_2_@TiO_2_ Nanoparticles

The core–shell SiO_2_@TiO_2_ nanoparticles were synthesized by sol–gel process according to the literature with minor modifications (Meng et al., 2012). Firstly, 0.3 g of SiO_2_ core spheres was sonicated in 100 mL of ethanol for 30 min to obtain solution A. Four milliliter of tetrabutyl titanate (TBT) was added in 100 mL of ethanol to obtain solution B. Then, solution B and 1.5 mL of aqueous ammonia were added to solution A under vigorous stirring at 60°C for 3 h. The resulting precipitates were centrifuged at 8,000 rpm and washed twice with ethanol and once with water. Finally, the obtained products were dried at 70°C for at least 20 h and were calcined at 850°C (10°C/min) for 2 h. Besides, the unsupported TiO_2_ was also synthesized using 4 mL of TBT by the same above procedures without SiO_2_ in the mixture.

### Synthesis of Hollow TiO_2_ Nanoparticles

Firstly, 0.5 g of calcined core–shell SiO_2_@TiO_2_ nanoparticles was dispersed in 60 mL of water under ultrasonication for 30 min. Subsequently, 3 mL of 2.5 M NaOH solution was added in the solution to etch the SiO_2_ core spheres from SiO_2_@TiO_2_ nanoparticles under vigorous stirring at 30°C for 6 h. Then, the etched hollow TiO_2_ nanoparticles were isolated by centrifugation at 8,000 rpm and washed twice with ethanol and once with water. Finally, the obtained hollow TiO_2_ nanoparticles were dried at 70°C for at least 20 h.

### Synthesis of ZIF-8

ZIF-8 was prepared according to the reported work (Liu et al., 2017). Typically, 0.595 g of Zn (NO_3_)_2_·6H_2_O (1 mM) and 1.314 g of 2-methylimidazole (8 mM) (Hmim) were dissolved in 40 mL of methanol separately. The Hmim solution was added slowly to the Zn^2+^ solution and then stirred at room temperature for 2 h. ZIF-8 was separated from the milky colloidal dispersion by centrifugation and washed with ethanol for three times, then dried at 70°C for 12 h.

### Synthesis of Double-Shell TiO2@ZIF-8 Hollow Nanoparticles

Firstly, each 0.3 g of synthesized hollow TiO_2_ nanoparticles was dispersed in 40 mL of methanol under ultrasonication for 30 min; meanwhile, different molar ratios of Zn (NO_3_)_2_·6H_2_O (0.5, 1, and 2 mM) were added to the above solution under stirring for 20 min. Secondly, different molar ratios of Hmim (4, 8, and 16 mM) were dissolved in 40 mL of methanol and stirred for 20 min, then the solutions were slowly added to the former mixture and stirred at room temperature for 2 h. The obtained precipitates were collected by repeated centrifugation with methanol for three times before drying at 70°C for 12 h. The synthesized double-shell hollow TiO_2_@ZIF-8 nanoparticles were labeled as HTZ-1, HTZ-2, and HTZ-3, respectively.

### Characterization

The JSM-6701F scanning electron microscope (SEM) was obtained. EDS was recorded using an energy-dispersive X-ray spectroscope (EDS) attached to the SEM. Transmission electron microscopy (TEM) was performed on a TECNAI G^2^. X-ray diffraction (XRD) patterns were estimated by a RINT 2000 diffractometer with Cu Ka radiation at 40 kV and 30 mA. X-ray photoelectron spectra (XPS) were collected using a PHI-5702 X-ray photoelectron spectroscope. N_2_ adsorption–desorption isotherms were investigated using an ASAP 2020 instrument surface area analyzer. Thermal gravimetric (TG) measurement was taken on a NETZSCHSTA 449 C with a heating rate of 10°C/min. UV-Vis diffuse reflectance spectra (DRS) of the samples were tested by a Lambda 950 UV-Vis spectrophotometer within a wavelength range of 200–800 nm, and BaSO_4_ was used as the reflectance standard.

### Photocatalytic Activity Experiments

The photocatalytic activities of all the synthesized samples were carried out by measuring the photodegradation of MB in a reactor under UV light irradiation with a 500-W high-pressure mercury lamp for 180 min. First, 75 mg of the synthesized photocatalysts was dispersed in 300 mL of MB aqueous solution (20 mg/L). Before photodegradation of MB, the reaction solution was magnetically stirred in the dark for 30 min to achieve adsorption–desorption equilibrium and then was carried out under UV light irradiation. During the photocatalytic degradation of MB in 180 min, 4 mL of the solution was collected from the reactor at different irradiation time intervals of every 20 min and then centrifuged. The concentration changes of MB were analyzed by recording the maximum absorbance of MB at 664 nm.

## Results and Discussion

### SEM and TEM Images, and EDX Spectrum

**Figure 1:**
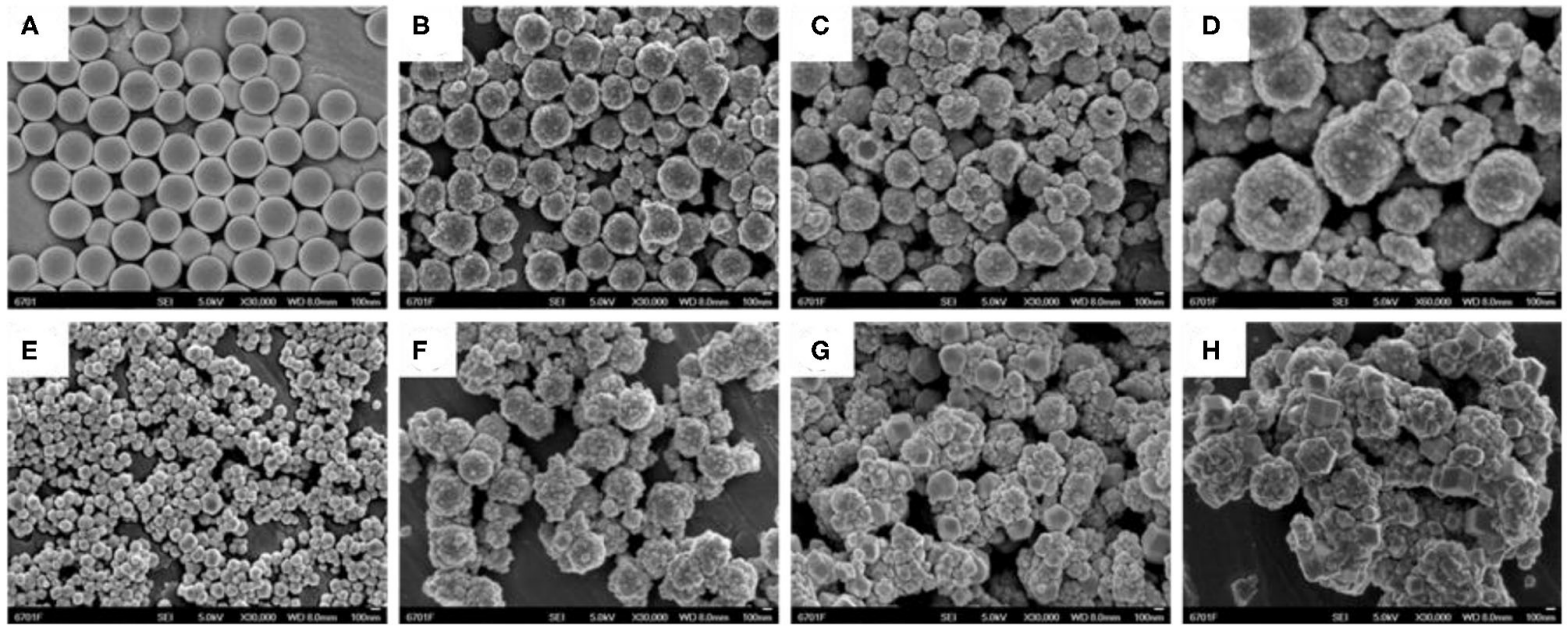
SEM images of **(A)** SiO_2_. **(B)** core-shell SiO_2_@TiO_2_ nanoparticles. **(C,D)** hollow TiO_2_ nanoparticles. **(E)** ZIF-8, **(F)** HTZ-1, **(G)** HTZ-2, and **(H)** HTZ-3.

**Figure 2:**
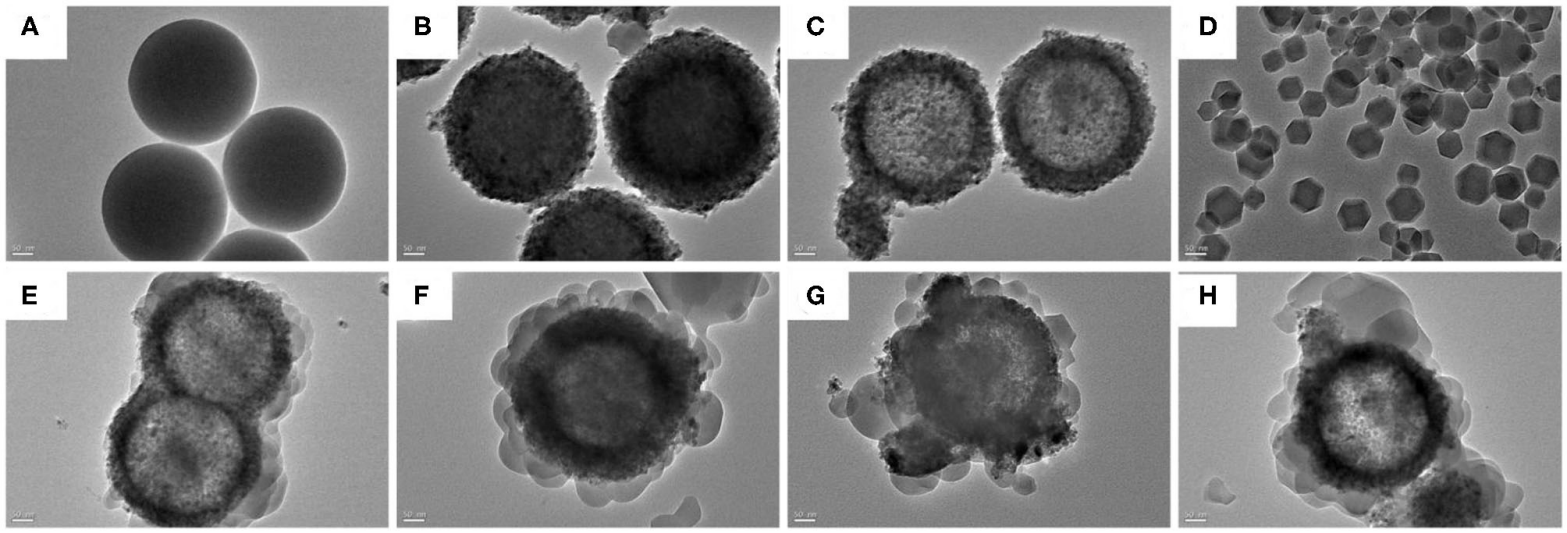
TEM images of **(A)** SiO_2_, **(B)** core-shell SiO_2_@TiO_2_ nanoparticles, **(C)** hollow TiO_2_ nanoparticles, **(D)** ZIF-8, **(E)** HTZ-1, **(F)**, **(G)** HTZ-2, and **(H)** HTZ-3.

Figures 1, 2 showed the morphology of synthesized samples. As shown in Figure 1A of SEM images and Figure 2A of TEM images, SiO_2_ spheres were spherical and smooth with a diameter of about 250–300 nm. The morphology of core–shell SiO_2_@TiO_2_ nanoparticles had rough and textured surfaces compared with SiO_2_ particles (Figure 1B), which indicated that TiO_2_ nanoparticles were coated on SiO_2_ and the core–shell structure of the nanoparticles formed by the sol–gel method as shown in Figure 2B. The spherical hollow TiO_2_ nanoparticles were obtained by etching SiO_2_ from core–shell SiO_2_@TiO_2_ nanoparticles, and the shell of TiO_2_ was about 30–50 nm (Figures 1C,D, 2C). The morphology of ZIF-8 was polyhedrons with a diameter of about 50–120 nm (Figures 1E, 2C). After synthesis of the hollow TiO_2_ nanoparticles with different molar ratios of ZIF-8, the ZIF-8 particles were successfully decorated onto hollow TiO_2_ nanoparticles to form the HTZ nanoparticles (Figures 1F–H). Compared with the SEM images of the HTZs, a more obvious double shell of the HTZ nanoparticles was shown in the TEM images and the content of the ZIF-8 particles increased in the double shell as the molar ratios of ZIF-8 increased (Figures 2E–H).

**Figure 3:**
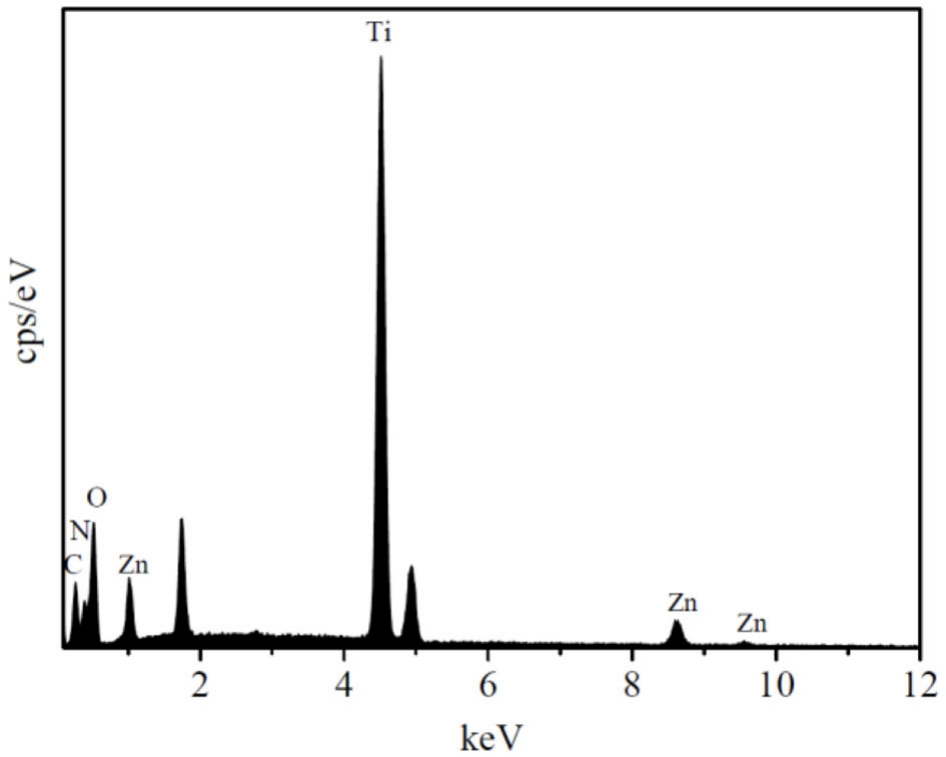
Energy-dispersive X-ray spectrometer (EDS) spectrum of the HTZ-2.

The EDS spectrum of the HTZ-2 nanoparticles is shown in Figure 3, which confirmed the presence of C, N, O, Ti, and Zn and indicated that the ZIF-8 particles were successfully decorated onto the HTZ nanoparticles.

### XRD Patterns

**Figure 4:**
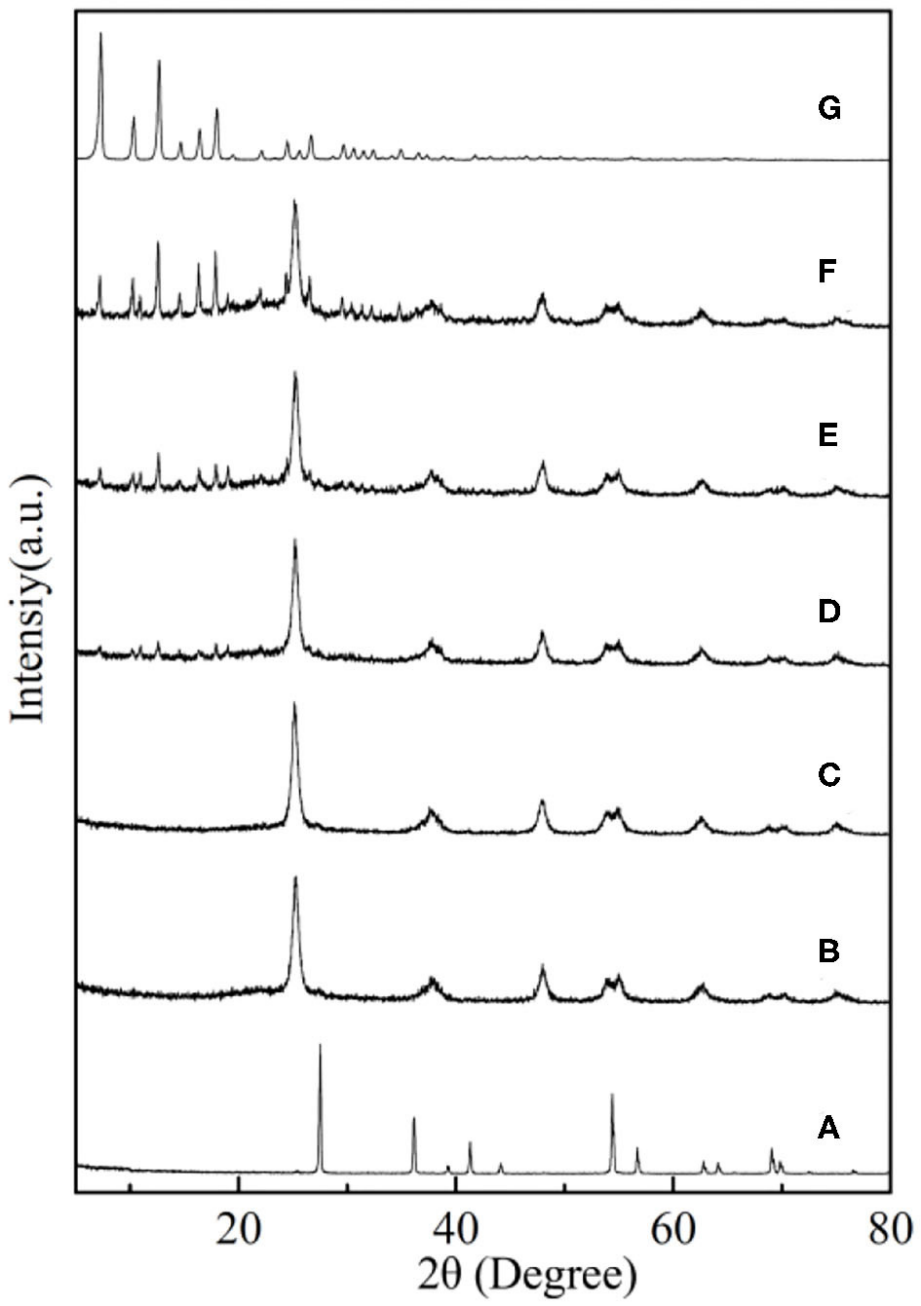
XRD patterns of **(A)** TiO_2_, **(B)** core–shell SiO_2_@TiO_2_ nanoparticles, **(C)** hollow TiO_2_ nanoparticles, and **(D)** HTZ-1, **(E)** HTZ-2, **(F)** HTZ-3, and **(G)** ZIF-8.

The XRD patterns of the synthesized nanoparticles are shown in Figure 4. As shown in Figure 4A, the TiO_2_ with 2θ peaks of 27.5, 36.2, 41.3, 54.4, and 69.0° was corresponded to the rutile phase (JCPDS No. 21-1276) after calcinated at 850°C (Periyat et al., 2008). However, the XRD patterns of core–shell SiO_2_@TiO_2_ nanoparticles with distinct peaks of 25.3, 37.9, 48.0, 62.7, and 75.0° were accorded with the anatase phase (JCPDS No. 21-1272) as shown in Figure 4B; this result indicated that the SiO_2_ core inhibited phase transformations of TiO_2_ from anatase to rutile at high calcination temperature of 850°C (Joo et al., 2012). The diffraction 2θ peaks of hollow TiO_2_ showed the same crystalline phase as core–shell SiO_2_@TiO_2_ nanoparticles and related to the anatase phase of TiO_2_ for just etching SiO_2_ by NaOH at room temperature. The XRD patterns of HTZ nanoparticles from Figure 4D to Figure 4F not only showed the anatase phase of TiO_2_ but also indicated the characteristic 2θ peaks of ZIF-8 at 7.4, 10.4, 12.7, 14.8, 16.5, and 18.0° (Figure 4G), which were consistent with the reported results (Zeng et al., 2016; Zhang et al., 2018). Besides, the 2θ peak intensity of ZIF-8 gradually became sharper and higher as the content of ZIF-8 increased in the HTZ nanoparticles, which also demonstrated that ZIF-8 was coated on hollow TiO_2_ and formed the double shell of hollow nanoparticles.

### XPS Analysis

**Figure 5:**
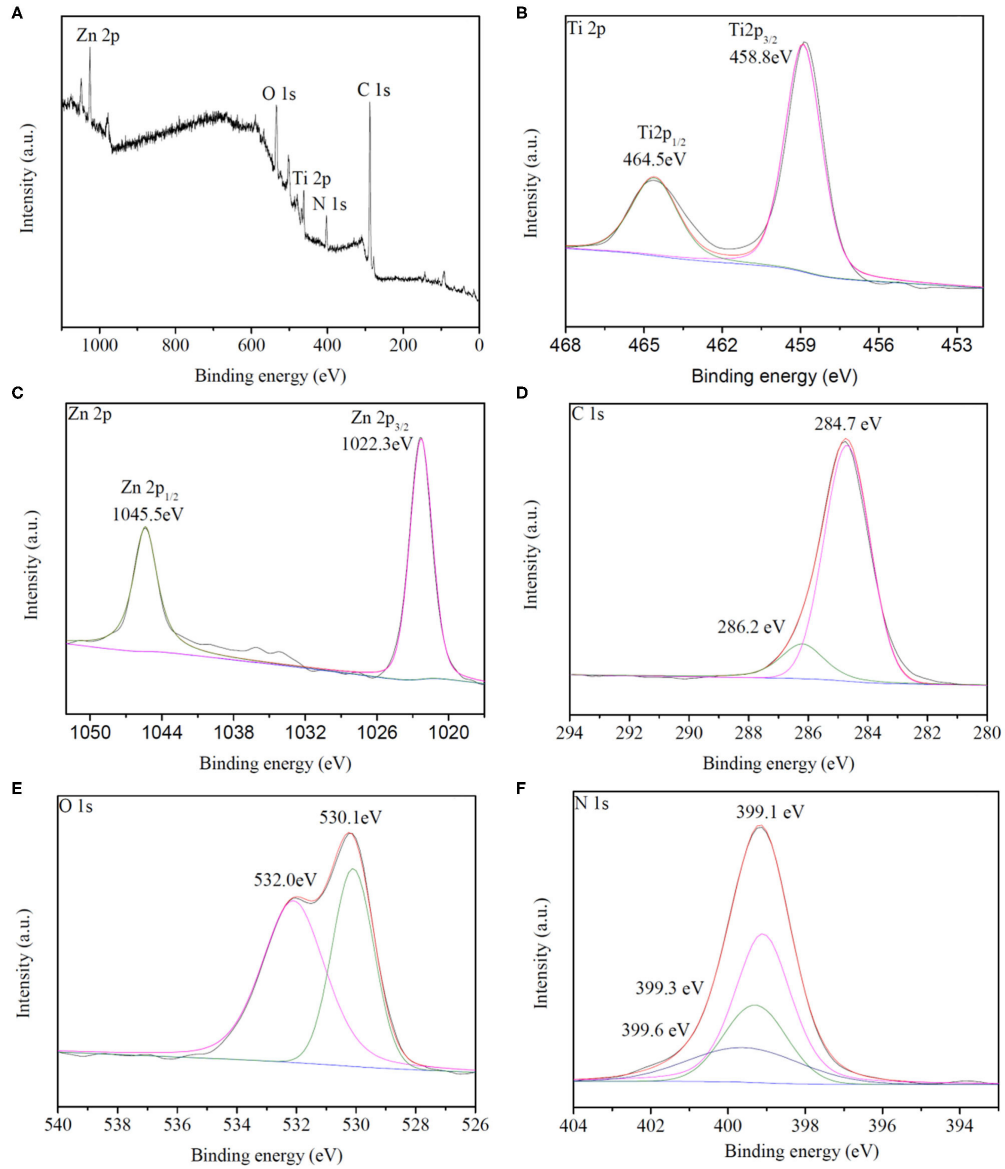
**(A)** XPS survey spectrum of HZT-2. **(B–F)** Ti 2p, Zn 2p, C 1s, O 1s, and N1s XPS spectrum of HZT-2.

X-ray photoelectron spectroscopy (XPS) was employed to analyze the chemical composition and valence state of the synthesized HTZ-2, as shown in Figure 5. The survey spectrum (Figure 5A) confirmed the elements of Ti, Zn, C, O, and N and the signals of Ti 2p, Zn 2p, C 1s, O 1s, and N 1s in HTZ-2. The XPS spectrum of Ti 2p (Figure 5B) showed the energy peak located at 458.8 and 464.5 eV, which were assigned to binding energies of Ti 2p 3/2 and Ti 2p 1/2 and corresponded to the presence of oxidation state Ti^4+^ in HTZ-2 (Chen et al., 2016; Wu et al., 2018). Figure 5C indicates that the binding energies of 1022.3 and 1045.3 eV for Zn 2p were attributed to Zn 2p 3/2 and Zn 2p 1/2, respectively (Tian et al., 2014). Figure 5D shows that the binding energies of 284.7 and 286.2 eV were assigned to the C–C and C–N bonds in the C 1 s spectrum (Wang et al., 2015). In Figure 5E, the peaks of O 1 s at 530.1 and 532.0 eV arose from the lattice oxygen in the TiO_2_ anatase phase and surface hydroxyl groups (Saliba et al., 2018). In the N 1 s XPS spectrum (Figure 5F), three peaks of 399.1, 399.3, and 399.6 eV were assigned to the N–C bond from imidazole groups in ZIF-8, N–H–bond, and N–Ti–O bond (Zhang et al., 2018). The presence of the N–Ti–O chemical bond in HTZ-2 indicated that part of O atoms was replaced by N atoms from imidazole groups on the surface of hollow TiO_2_ and that ZIF-8 was synthesized successfully with hollow TiO_2_, which formed the heterostructure of double shell hollow nanoparticles, as also shown in SEM and TEM images (Zeng et al., 2016).

### TG Analysis

**Figure 6:**
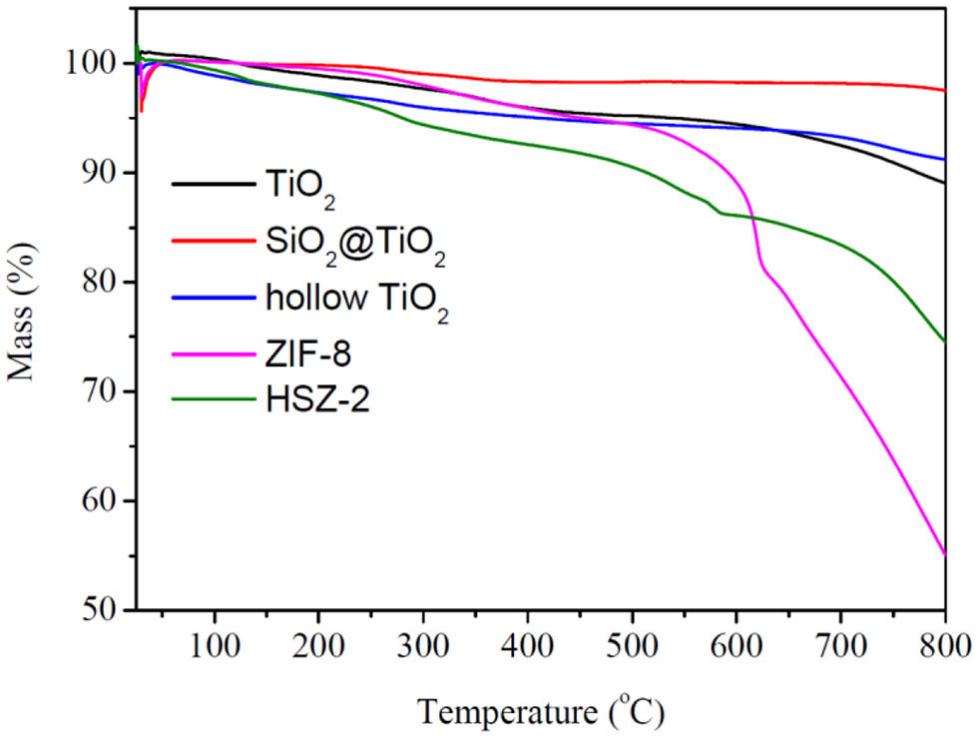
TG analysis of TiO_2_, core–shell SiO_2_@TiO_2_ nanoparticles, hollow TiO_2_ nanoparticles, ZIF-8, and HTZ-2.

The thermal gravimetric (TG) analysis was processed to evaluate the thermal stability of the prepared samples. As shown in Figure 6, TiO_2_, core–shell SiO_2_@TiO_2_ nanoparticles, and hollow TiO_2_ nanoparticles showed no more than mass loss of 10% up to 800°C. However, for bare ZIF-8, there was no obvious mass loss up to 600°C; a total mass loss of 44.89% occurred in the temperature range from 600 to 800°C, which attributed to the collapse of the ZIF-8 structure. For HZT-2, the TG analysis exhibited a total mass loss of 25.50% up to 800°C; it was indicated that the ZIF-8 shell in the hybrid structure showed certain thermal stability and the decomposition of the HZT occurred just at high temperature (Zhang et al., 2018).

### N_2_ Adsorption–Desorption Isotherm Analysis

**Figure 7:**
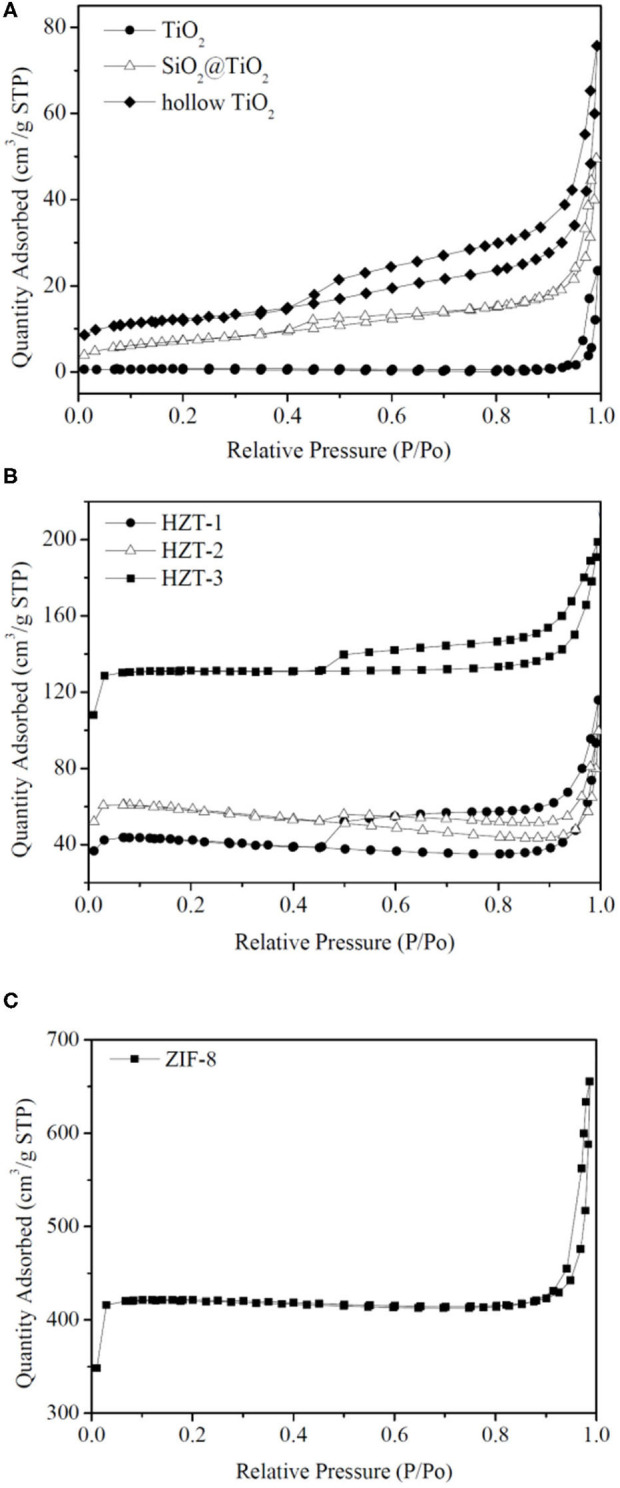
**(A–C)** N_2_ adsorption–desorption isotherm analysis of the synthesized nanoparticles.

Figure 7 shows the nitrogen adsorption–desorption isotherms, and Table 1 indicates the Brunauer–Emmett–Teller (BET) surface area and pore size parameters of the synthesized samples. As shown in Figure 7A, the adsorption isotherm of TiO_2_ nanoparticles can be categorized as type I with microporous characteristic, which was caused by calcinating at 850°C and the growth of the particle size (Periyat et al., 2008). However, compared with TiO_2_, the adsorption isotherm of hollow TiO_2_ nanoparticles exhibited the shape of type IV with H2 hysteresis loops and indicated them to be mesoporous materials (Liu et al., 2017). In addition, the specific BET surface area of hollow TiO_2_ was 40.77 m^2^g^−1^, which was more than 15 times higher than the TiO_2_ nanoparticles (2.62 m^2^g^−1^) and nearly 2 times higher than the core–shell SiO_2_@TiO_2_ nanoparticles (25.88 m^2^g^−1^).

**Table 1:** BET surface area and pore size parameters of the synthesized nanoparticles.

**Samples**	**BET surface area (m^**2**^g^**−1**^)**	**Pore size (nm)**	**Pore volume (cm^**3**^ g^**−1**^)**
TiO_2_	2.62	65.0	0.04
SiO_2_@ TiO_2_	25.88	0.08	11.9
hollow TiO_2_	40.77	0.11	14.0
HZT-1	118.48	0.13	70.0
HZT-2	158.42	0.091	92.4
HZT-3	389.07	0.11	37.9
ZIF-8	1245	0.38	55.0

Figure 7C shows that ZIF-8 had the nitrogen adsorption–desorption isotherm of type I and fitted well with the microporous frameworks of ZIF-8 (Zeng et al., 2016). Besides, the hysteresis loop of ZIF-8 at high relative pressure (0.9–1.0) indicated the existence of textural macroporosity formed by packing of ZIF-8 crystals and the high surface area of 1,245 m^2^g^−1^, according to reported studies (Zeng et al., 2016; Liu et al., 2017). After synthesizing the hollow TiO_2_ with ZIF-8 and forming HZTs, as shown in Figure 7B, the nitrogen adsorption–desorption isotherm of HZTs exhibited the shape of type IV with H2 hysteresis loops and indicated them to be mesoporous materials. The surface area of HZTs was higher than the core–shell SiO_2_@TiO_2_ and hollow TiO_2_, which also gradually increased as the content of ZIF-8 increased, as shown in Table 1.

### UV-Vis Diffuse Reflectance Spectroscopy

**Figure 8:**
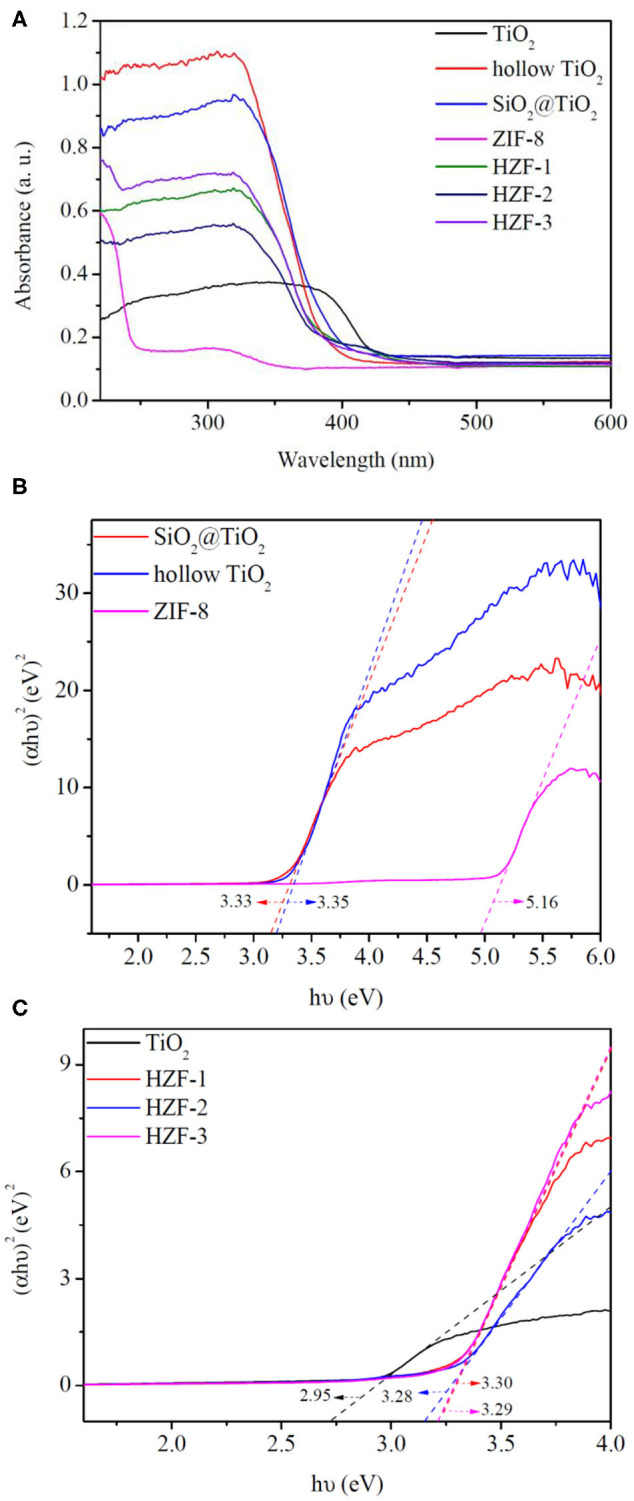
**(A)** UV-visible absorption spectra of the synthesized nanoparticles. **(B,C)** Fit curves of Tauc plot for band gap of the synthesized nanoparticles.

The optical property of the synthesized samples was evaluated by UV-Vis diffuse reflectance spectroscopy (DRS). The optical adsorption spectra of the nanocomposite samples are shown in Figure 8A; ZIF-8 showed an absorption edge <240 nm. TiO_2_ had an absorption band in the 380–430 nm region because of the crystal structure in the rutile phase. The core–shell SiO_2_@TiO_2_ nanoparticles and hollow TiO_2_ nanoparticles showed the same absorption band around 320–420 nm. The absorption band of the HZTs had a blue shift to the UV absorption region in the range of 320–400 nm for the synergistic effect between hollow TiO_2_ and ZIF-8.

The optical band gaps (Eg) of the synthesized nanoparticles were estimated by the Tauc plot as shown in Figures 8B,C; the band gaps of core–shell SiO_2_@TiO_2_ nanoparticles, hollow TiO_2_ nanoparticles, and ZIF-8 were evaluated to be 3.33, 3.35, and 5.16 eV. The band gap of TiO_2_ nanoparticles was 2.95 eV, which was aroused from the rutile phase similarly with the absorption band. The band gaps of HZT-1, HZT-2, and HZT-3 were about 3.30, 3.28, and 3.29 eV, respectively. HZT-2 showed relatively the lowest band gaps in the HZTs nanoparticles; the results indicated that the ZIF-8 coating was beneficial to the charge separation and the decrease in Eg was helpful in enhancing the photocatalytic efficiency for promoting the recombination of the photo-generated electron-hole pairs (Choi et al., 2010; Zhang et al., 2018).

### Photocatalytic Activity

**Figure 9:**
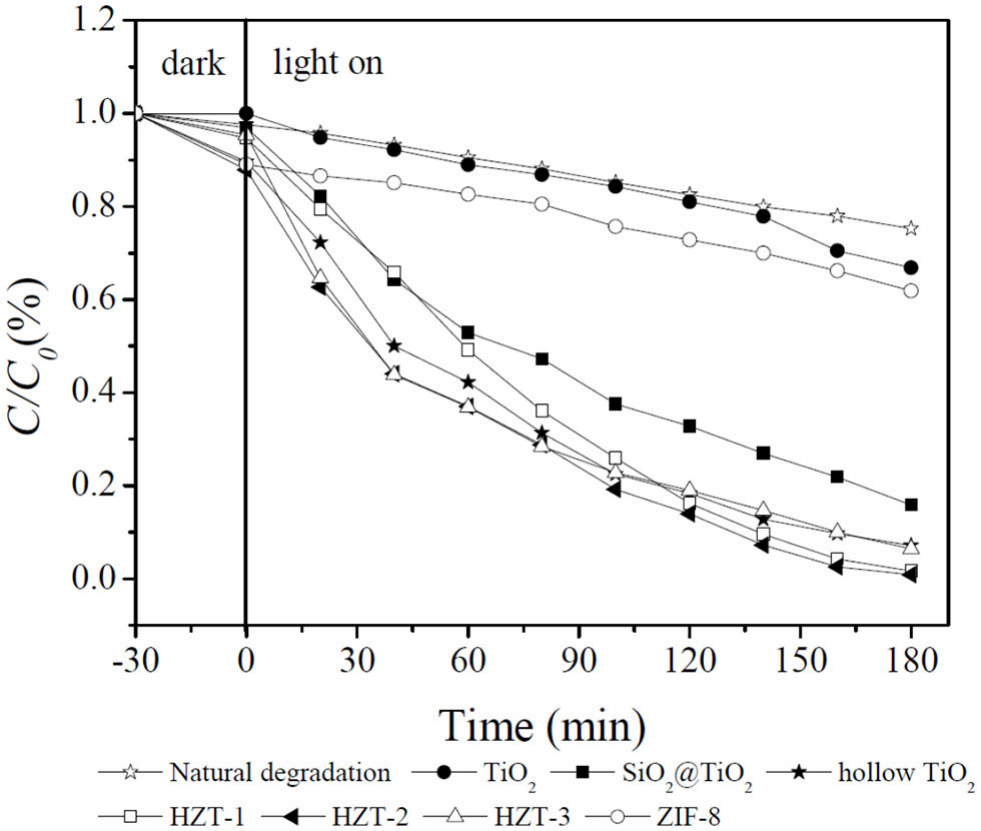
Photodegradation of MB using synthesized nanocomposites.

The photocatalytic activity of the synthesized nanocomposites for the degradation of MB under UV light irradiation is studied in Figure 9. As shown in Figure 9, TiO_2_ had poor photocatalytic activity of 33.2% because of the rutile phase after calcination at 850°C for 2 h. ZIF-8 also showed poor photocatalytic activity of 38.1% under UV irradiation due to its large band gap of 5.16 eV (Zhang et al., 2018). The core–shell SiO_2_@TiO_2_ nanoparticles had higher photocatalytic activity of 84.1% than TiO_2_. Generally, hollow TiO_2_ nanoparticles and HZTs showed higher photocatalytic activity than core–shell SiO_2_@TiO_2_ and TiO_2_ nanoparticles. The photocatalytic efficiency followed the order HZT-2 > HZT-1 > HZT-3 > hollow TiO_2_ nanoparticles > SiO_2_@TiO_2_ nanoparticles > ZIF-8 > TiO_2_. The hybrid of ZIF-8 and hollow TiO_2_ nanoparticles can promote the photocatalytic activity, and HZT-2 had the highest photocatalytic efficiency of 99.1% toward MB under UV light. There were several reasons for the high photocatalytic activity of HZTs. Firstly, the inner hollow TiO_2_ nanoparticles supplied a higher specific surface area and more active sites to improve the photocatalytic activity. However, the HZT nanoparticles synthesized by hollow TiO_2_ and ZIF-8 showed a much higher specific surface area than hollow TiO_2_ nanoparticles, which was attributed to the properties of ZIF-8 (Li et al., 2011; Chandra et al., 2016). Secondly, the synergistic effect of the heterostructure between hollow TiO_2_ and ZIF-8 narrowed the band gap and improved the photoelectron transfer to the surface of hollow TiO_2_, as shown in Figure 8. Thirdly, just a proper content of ZIF-8 coated on hollow TiO_2_ can be beneficial for separating the charge and increase the radical oxide O^2−^ generated from HZT nanoparticles for the photocatalytic degradation of MB (Cong et al., 2007; Zhang et al., 2018). Furthermore, the chemical bonding structure of the HZT nanoparticles can improve the separation efficiency of the photoelectron pairs and accelerate the photocatalytic efficiency (Zeng et al., 2016).

**Figure 10:**
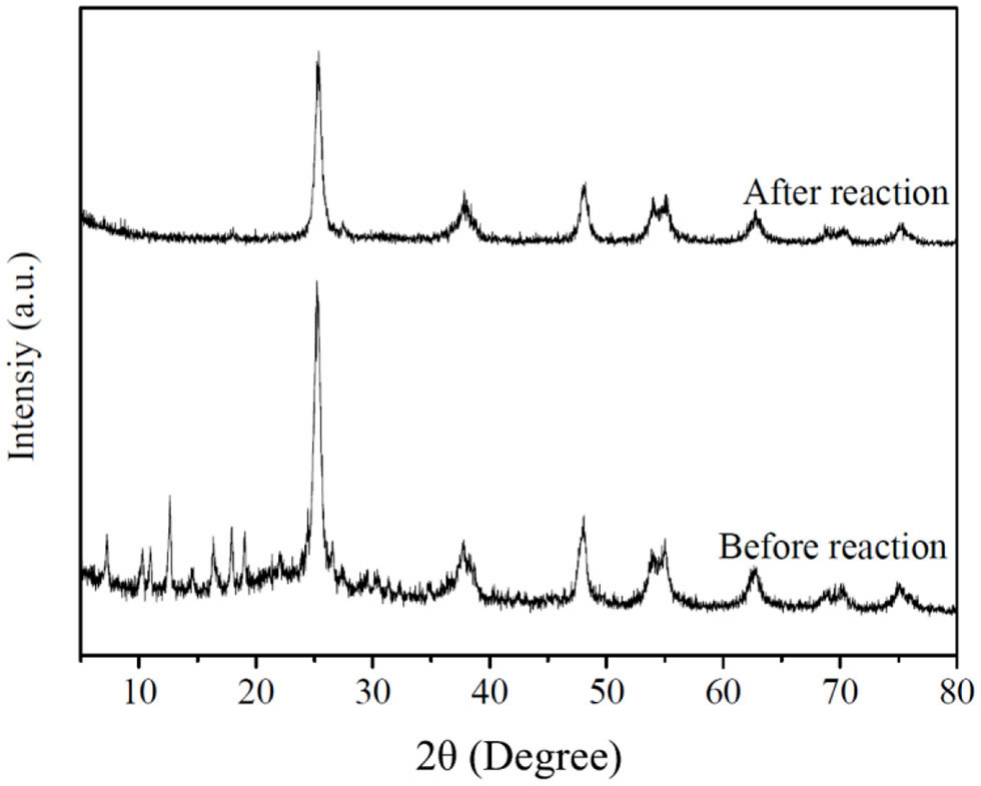
XRD of HZT-2 before and after reaction.

The XRD of HZT-2 before and after the photocatalytic experiment was processed in Figure 10. As shown in Figure 10, after reaction the XRD of HZT-2 indicated that TiO_2_ still kept the anatase crystal structure, but ZIF-8 decreased the crystal structure and the characteristic diffraction peak of ZIF-8 was not obvious compared with the XRD of HZT-2 before reaction. This indicated that the structure of the HZT-2 photocatalyst decomposed under UV light.

## Conclusion

In this study, the double-shell hollow TiO_2_@ZIF-8 nanoparticles were synthesized by coating ZIF-8 on the hollow TiO_2_ nanoparticles. The morphology and other properties of the synthesized nanoparticles were studied by SEM, TEM, EDX, XRD, XPS, and BET surface area, TG, and UV-Vis. TiO_2_ was indicated to be in the rutile phase after being calcined at 850°C. However, the core–shell SiO_2_@TiO_2_ nanoparticles and hollow TiO_2_ nanoparticles showed to be in the anatase phase. The HTZ nanoparticles not only showed the anatase phase of TiO_2_ but also indicated the characteristic peaks of ZIF-8.

The specific surface area of the HTZs was significantly increased by coating ZIF-8, and the HTZ nanoparticles exhibited a mesoporous structure with the shape of type IV and H2 hysteresis loops. The TG analysis showed that bare ZIF-8 had a total mass loss of 44.89% and HZT-2 exhibited a total mass loss of 25.50% up to 800°C. The UV-Vis results indicated that the coating of ZIF-8 narrowed the band gap of the HZTs compared with the core–shell SiO_2_@TiO_2_ nanoparticles and hollow TiO_2_. The photocatalytic activity indicated that HTZ nanoparticles showed higher photodegradation efficiency due to the synergistic effect of the heterostructure toward MB under UV light irradiation. The highest photocatalytic activity of 99.1% was obtained from HZT-2, which was synthesized with proper content of ZIF-8 adding on hollow TiO_2_ toward MB. This work indicates that the heterostructure of hollow TiO_2_ and ZIF-8 is a promising photocatalyst for the treatment of dye pollutants in wastewater.

## Data Availability Statement

All datasets generated for this study are included in the article/Supplementary Material.

## Author Contributions

X-cR conceived and supervised the project. NF performed the experiment, analyzed the data, and wrote the manuscript. All authors discussed the results and commented on the paper. All authors contributed to the article and approved the submitted version.

## Conflict of Interest

The authors declare that the research was conducted in the absence of any commercial or financial relationships that could be construed as a potential conflict of interest.
