# Supplementary figures and images for: Synthesis of Double-Shell Hollow TiO2@ZIF-8 Nanoparticles With Enhanced Photocatalytic Activities
Source: Front Chem. 2020 Oct 23;8:578847. doi: 10.3389/fchem.2020.578847 (PMC7645166; doi:10.3389/fchem.2020.578847)

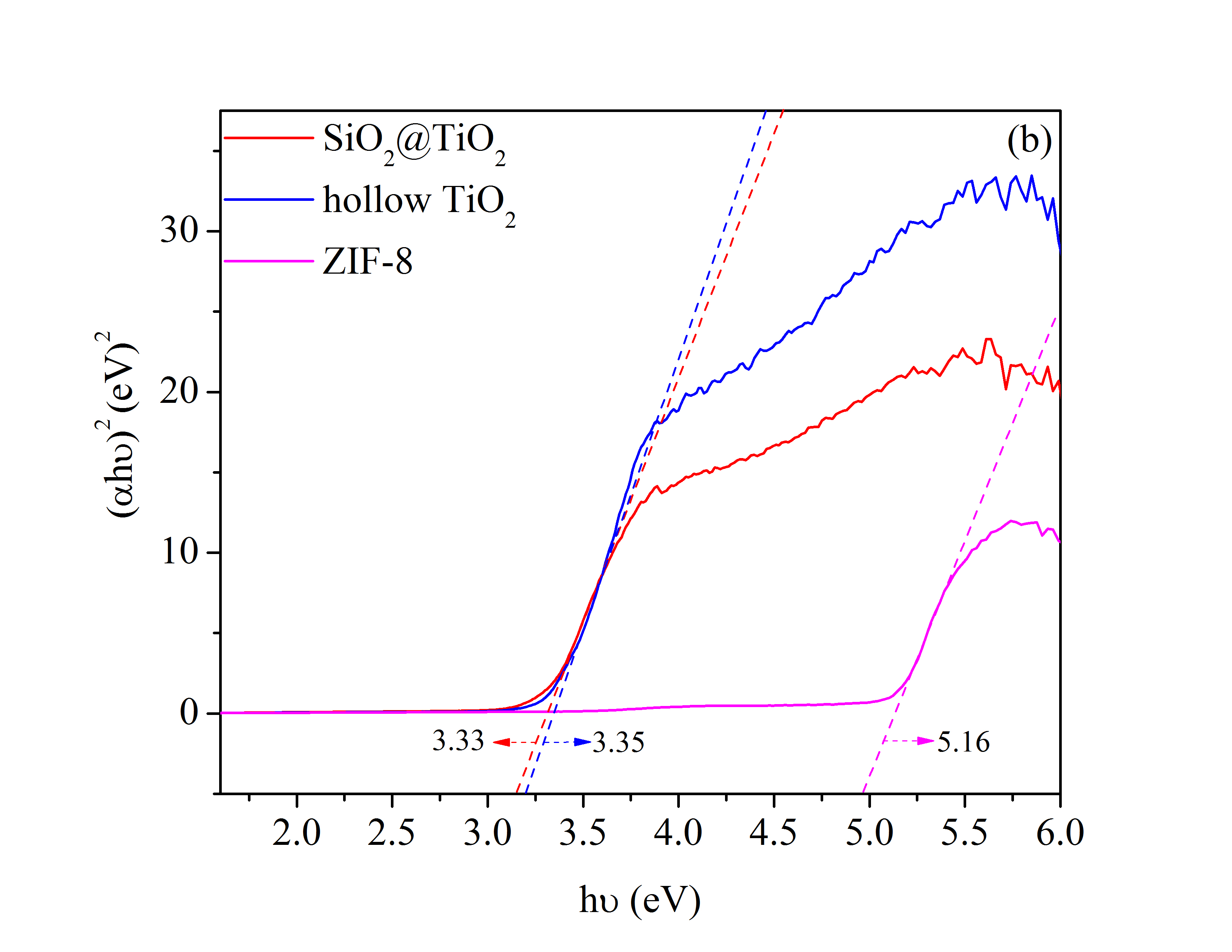

Supplement: Supplementary file 1 [file Image_1.JPEG]

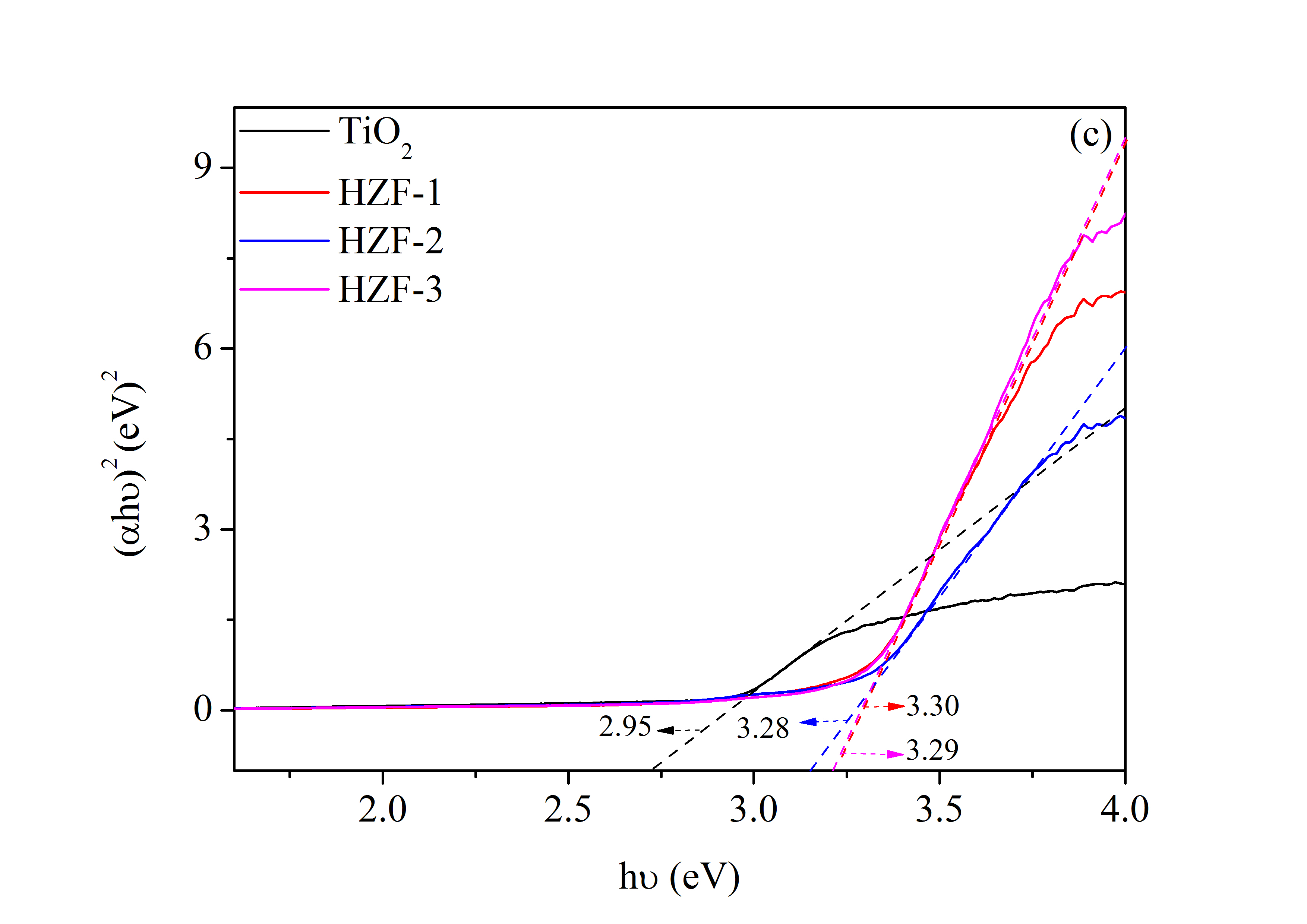

Supplement: Supplementary file 2 [file Image_2.JPEG]

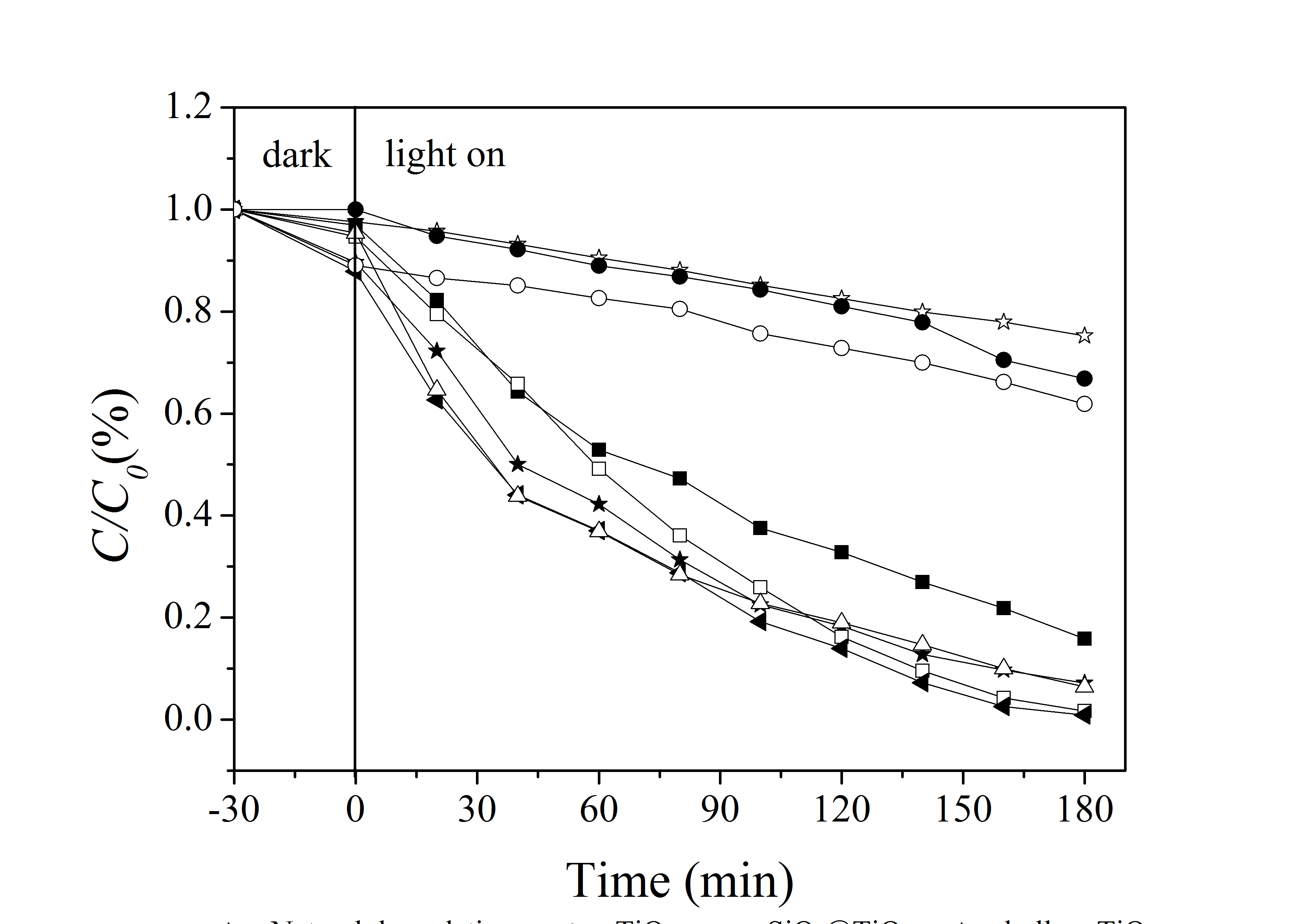

Supplement: Supplementary file 3 [file Image_3.JPEG]

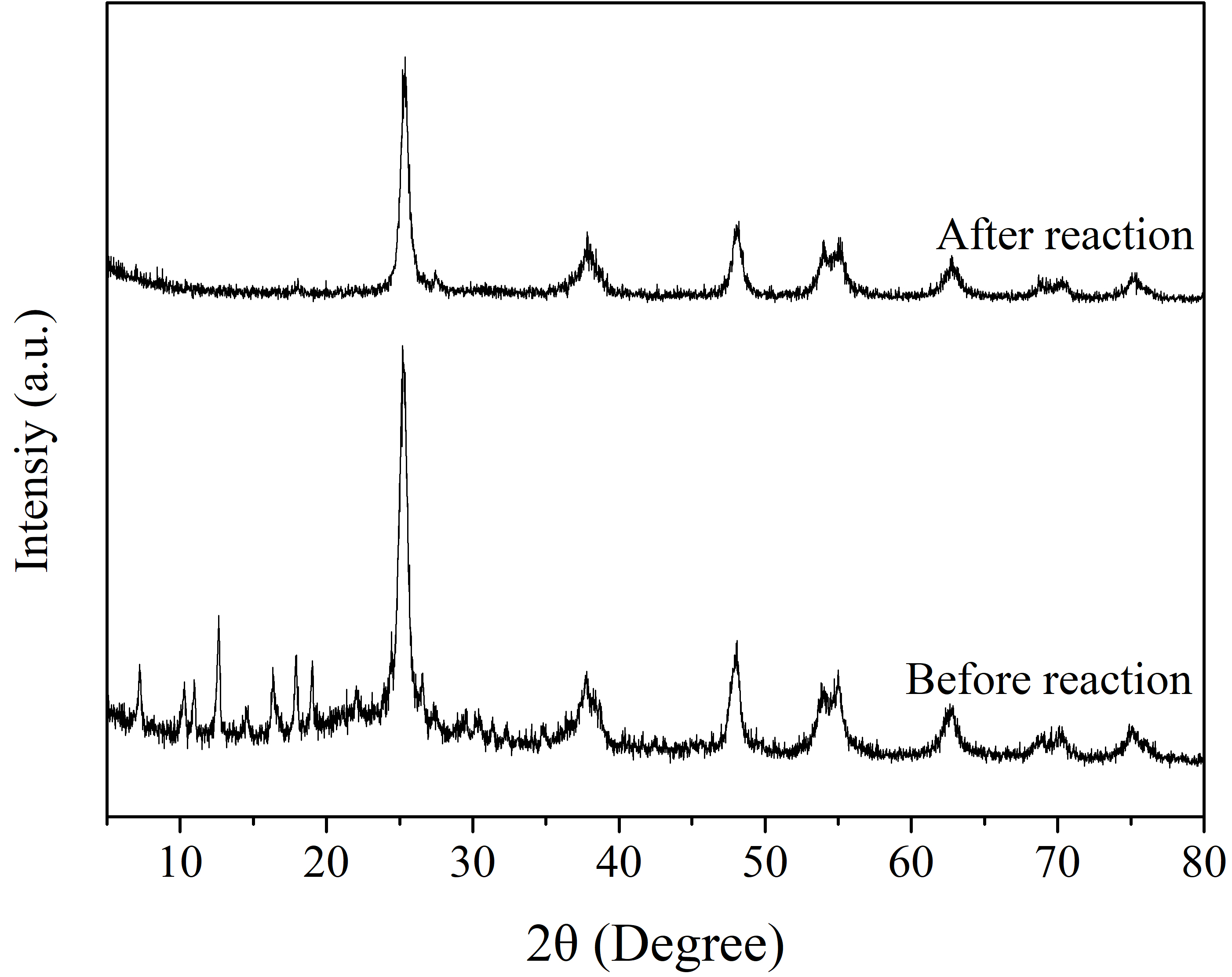

Supplement: Supplementary file 4 [file Image_4.JPEG]
